# Digital Pills to Measure Opioid Ingestion Patterns in Emergency Department Patients With Acute Fracture Pain: A Pilot Study

**DOI:** 10.2196/jmir.7050

**Published:** 2017-01-13

**Authors:** Peter R Chai, Stephanie Carreiro, Brendan J Innes, Rochelle K Rosen, Conall O'Cleirigh, Kenneth H Mayer, Edward W Boyer

**Affiliations:** ^1^ Division of Medical Toxicology Department of Emergency Medicine Brigham and Women's Hospital Boston, MA United States; ^2^ Division of Medical Toxicology Department of Emergency Medicine University of Massachusetts Medical School Worcester, MA United States; ^3^ University of Massachusetts Medical School Worcester, MA United States; ^4^ Behavioral and Preventative Medicine The Miriam Hospital Brown School of Public Health Providence, RI United States; ^5^ Department of Psychiatry Massachusetts General Hospital Boston, MA United States; ^6^ Department of Medicine Beth Israel Deaconess Medical Center Harvard Medical School Boston, MA United States

**Keywords:** medication adherence, opioid, digital pills, digital health, emergency medicine, pain management

## Abstract

**Background:**

Nonadherence to prescribed regimens for opioid analgesic agents contributes to increasing opioid abuse and overdose death. Opioids are frequently prescribed on an as-needed basis, placing the responsibility to determine opioid dose and frequency with the patient. There is wide variability in physician prescribing patterns because of the lack of data describing how patients actually use as-needed opioid analgesics. Digital pill systems have a radiofrequency emitter that directly measures medication ingestion events, and they provide an opportunity to discover the dose, timing, and duration of opioid therapy.

**Objective:**

The purpose of this study was to determine the feasibility of a novel digital pill system to measure as-needed opioid ingestion patterns in patients discharged from the emergency department (ED) after an acute bony fracture.

**Methods:**

We used a digital pill with individuals who presented to a teaching hospital ED with an acute extremity fracture. The digital pill consisted of a digital radiofrequency emitter within a standard gelatin capsule that encapsulated an oxycodone tablet. When ingested, the gastric chloride ion gradient activated the digital pill, transmitting a radiofrequency signal that was received by a hip-worn receiver, which then transmitted the ingestion data to a cloud-based server. After a brief, hands-on training session in the ED, study participants were discharged home and used the digital pill system to ingest oxycodone prescribed as needed for pain for one week. We conducted pill counts to verify digital pill data and open-ended interviews with participants at their follow-up appointment with orthopedics or at one week after enrollment in the study to determine the knowledge, attitudes, beliefs, and practices regarding digital pills. We analyzed open-ended interviews using applied thematic analysis.

**Results:**

We recruited 10 study participants and recorded 96 ingestion events (87.3%, 96/110 accuracy). Study participants reported being able to operate all aspects of the digital pill system after their training. Two participants stopped using the digital pill, reporting they were in too much pain to focus on the novel technology. The digital pill system detected multiple simultaneous ingestion events by the digital pill system. Participants ingested a mean 8 (SD 5) digital pills during the study period and four participants continued on opioids at the end of the study period. After interacting with the digital pill system in the real world, participants found the system highly acceptable (80%, 8/10) and reported a willingness to continue to use a digital pill to improve medication adherence monitoring (90%, 9/10).

**Conclusions:**

The digital pill is a feasible method to measure real-time opioid ingestion patterns in individuals with acute pain and to develop real-time interventions if opioid abuse is detected. Deploying digital pills is possible through the ED with a short instructional course. Patients who used the digital pill accepted the technology.

## Introduction

Deaths from opioid overdose in the United States have paralleled a rise in the number of opioid analgesic medications being prescribed [1]. In 2014, more than 259 million prescriptions for opioids were dispensed to the American public [1-3]. Opioids are commonly prescribed on an as-needed basis, meaning that decisions regarding opioid dose and frequency are left up to patients. Unfortunately, the ways in which patients use as-needed opioids outside of hospital settings are unknown, as is the optimal duration of opioid therapy needed for acutely painful conditions. This uncertainty has contributed to wide variability in opioid-prescribing patterns, often for an excessive number of opioid pills than what are actually needed [2,4-6].

Determining adherence to a prescribed opioid medication regimen after discharge and measuring the ways in which patients ingest as-needed medications pose methodological challenges. Because patients determine opioid ingestion dose and frequency based on temporal perception of pain, common measures of adherence (eg, smart pill bottles, pharmacy refill histories, or patient diaries) are impractical or provide aggregate measures that cannot delineate temporal patterns of opioid ingestion [7]. These traditional models measure adherence indirectly and are fraught with recall bias, measure ingestions in aggregate, and are easy to subvert (eg, opening the bottle once, but taking out several pills) [7,8]. Precise measures of opioid ingestion patterns are important because they can suggest incomplete treatment of pain, the development of tolerance, or the transition into problematic use [9].

### A Novel Digital Pill System to Measure Medication Ingestion Patterns

**Figure 1:**
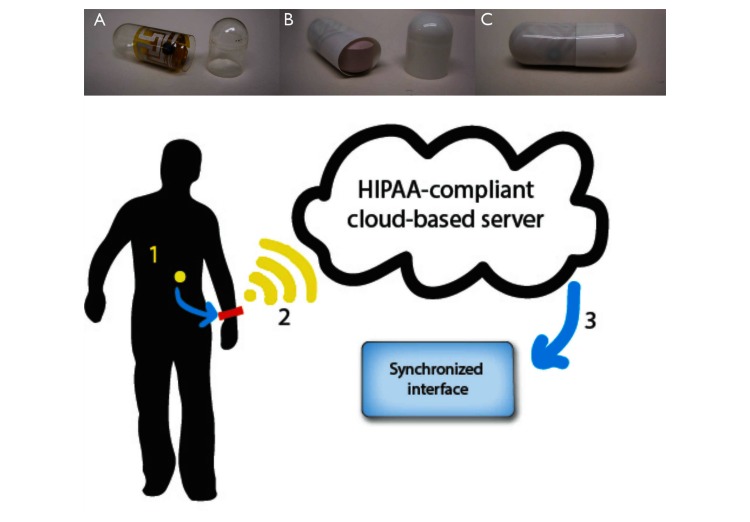
An ingestible radiofrequency sensor is incorporated into a gelatin capsule (A), which is compounded with the desired medication (B) to create a digital pill (C). Once the digital pill is ingested (1), it is activated and transmits a radiofrequency signal to a hip worn device (2) that collects and transmits ingestion data to a cloud-based server driving an interface (3) that displays ingestion data to clinicians and patients.

Digital pills that activate upon contact with the stomach provide a reliable method to directly measure, rather than infer, opioid ingestion patterns in real time [10,11]. Digital pills consist of a gelatin capsule containing a digital radiofrequency emitter compounded with the desired medication (Figure 1) [10,12]. When ingested, the radiofrequency emitter is activated by the chloride ion gradient in the stomach to transmit a unique signal. A hip-worn receiver detects this signal before relaying data regarding the identity of the ingested medication and time of ingestion through third-generation (3G) cellular signaling to a cloud-based server compliant with Health Information Technology for Economic and Clinical Health (HITECH) and the Health Insurance Portability and Accountability Act (HIPAA). A locus for communication, the server can notify clinicians about the ingestion, send messages that can include behavioral interventions to the patient, and facilitate communication between clinician and patient. Digital pills are energized by the specific chloride ion gradient in the stomach and, therefore, cannot be activated outside of the body. Because each digital pill emits a unique frequency, the system can record multiple simultaneous ingestion events. This technological advantage, which allows the direct observation of the number of opioid pills a patient ingests as well as the time period between opioid ingestion, can personalize the number of opioid pills and the duration of as-needed opioid therapy [12]. Digital pills are simple to operate and passively measure adherence, decreasing the need for an individual to interact with technology to transmit adherence data. Unfortunately, the usability and acceptability of digital pills among “real-world” patient populations remains unknown. Accordingly, we sought to determine the acceptability of digital pills to emergency department (ED) patients with acute extremity fractures instructed to ingest oxycodone prescribed on an as-needed basis.

## Methods

Our study was based at a large tertiary care academic ED, and approved by its institutional review board (University of Massachusetts Medical School, Worcester, MA, USA). We used a digital pill (eTectRx, Newbury, FL, USA) compounded with oxycodone tablets in patients who presented to the ED after suffering an acute extremity fracture to measure real-world ingestion patterns in these patients. Digital pills were purchased directly from the manufacturer (eTectRx) and compounded with 5 mg oxycodone tablets using a standard capsule-filling machine by our hospital investigational drug services pharmacy. Compounded digital pills were dispensed in blister packages. We considered alternative digital pill systems that used radiofrequency signaling to detect ingestion events. Other digital pills required the radiofrequency emitter to be adhered onto a placebo pill, thus requiring ingestion of the study medication and placebo pill to record an ingestion event. We elected to utilize the eTectRx system because it offered the opportunity to directly measure, rather than infer, medication ingestion.

### Recruitment

A convenience sample of ED patients was recruited during daytime hours when research staff was available. Patients were eligible for the study if they were consenting adults (>18 years); presenting to the ED after an isolated acute fracture; had no history of psychiatric illness, substance abuse disorder, or chronic opioid use (prescribed opioids for more than one week in duration); and were planned to be discharged with opioid analgesics. Patients who met the eligibility criteria were approached by study staff. A chart review was conducted to ensure potential study participants did not meet exclusion criteria. Basic demographic information including age, gender, and ED pain scale were collected. Study staff then briefed potential participants on the purpose of the study and potential study participants were shown components of the digital pill. Participants who agreed to participate in the study provided written informed consent. Participants were compensated US $100 for participation in the study.

### Training to Use the Digital Pill

Study staff provided participants with hands-on training lasting approximately 20 minutes on the operation of the digital pill. Training included a demonstration of digital pill operations, explanation of all the hardware components, and registration of the study participant on the cloud interface. Participants were counseled by the study staff to use nonopioid analgesics (nonsteroidal anti-inflammatory agents or acetaminophen) to manage their pain, and use oxycodone-containing digital pills as needed for episodes of pain not controlled with other medications. Participants then ingested an oxycodone-containing digital pill under observation by study staff to demonstrate their understanding of the system and to verify that all components of the system were operational. If a patient reported their pain levels were managed during the time of system training, participants demonstrated their understanding of the system without ingesting an oxycodone pill. On discharge, participants received a prescription for one-to-two digital pills containing 5 mg oxycodone every 8 hours as needed for pain (total of 21 digital pills) in addition to a hip-worn device to capture ingestion data. Study participants enrolled in the study did not receive additional opioid analgesics from the primary team.

### Qualitative Analysis and Verification of Ingestion

Participants returned to the hospital’s clinical research center at the time of their orthopedic follow-up appointment or at one week after ED discharge to return the digital pill system and any uningested oxycodone digital pills. Equipment return and appointments were arranged with the study participants by study staff. We validated ingestion data from the digital pill using pill counts; we resolved discrepancies, if present, with the participant.

Investigators (PRC, EWB, BI) conducted brief (approximately 15 minutes), open-ended interviews with each study participant. We used an interview guide to ensure that each participant was asked the same questions; questions centered on acceptance of the digital pill, barriers to use, adequacy of pain control, and potential improvements in the system. We adapted the technique of applied thematic analysis to code interviews in the context of this pilot study [13]. Participant responses were transcribed by BI and reviewed by the three qualitative analysts (PRC, BI, and EWB), who then met to review topics. Deductive codes were created based on the key interview questions (acceptance of digital pills, willingness to use digital pills, and data privacy); additional inductive codes were derived from emergent participant comments (ie, willingness to use during chronic care, text message reminders, data security, and privacy). After coding, two of the investigators (PRC and BI) met and compared codes to ensure interrater reliability. Summaries of key codes were written and reviewed among the investigators. In this limited and exploratory sample, a key concern was to capture both the common and uncommon experiences participants reported with the pill and sensor.

## Results

**Figure 2:**
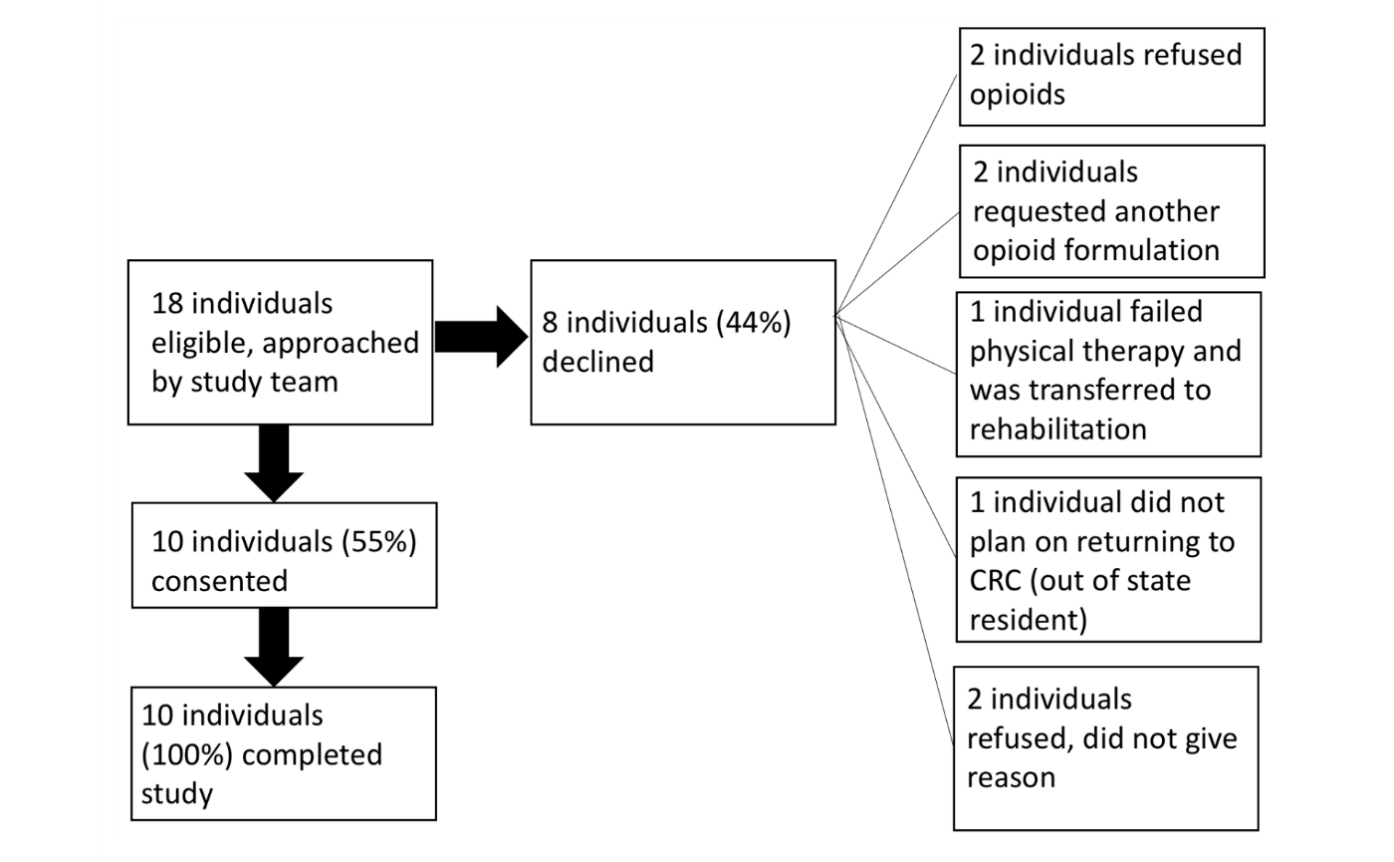
Study enrollment schema.

Eighteen individuals met the inclusion criteria for the study and were approached by the study team (Figure 2). Ten individuals ultimately consented and enrolled in the study (Table 1). They were evenly split between men and women and ranged in age from 21 to 63 years, with a mean age of 43 (SD 15) years. All participants successfully completed training in the ED and used the digital pill system at home. All individuals reported severe pain (pain scale ≥7) on initial presentation to the ED. Five participants required delayed surgical management of their fracture and five participants were managed nonoperatively.

Participants ingested a mean 48 (SD 24) mg oxycodone during the course of the study (Table 2). Oxycodone was ingested in greater frequency during the first 24 hours after discharge, with declining use by 72 hours. Seven participants de-escalated their oxycodone use over two days, changing dosing frequency (from every 4-6 hours to every 8-12 hours) in conjunction with dosing amount (from 10 mg oxycodone per ingestion to 5 mg oxycodone per ingestion). One participant experienced pain from associated injuries and increased the oxycodone dose over the study period. Four participants reported requiring continued opioid analgesia at the end of the study period (40%, 4/10); these participants all required operative repair of their fracture. No participants with nonoperative fractures reported persistent opioid use at the end of the study period.

**Table 1:** Demographics, fracture pattern, and final treatment of study participants (N=10)

Participant	Age (years)	Sex	Injury	Definitive therapy	Time until orthopedic follow-up (days)
1	32	M	Lisfranc fracture	Surgical fixation	3
2	48	F	Distal radius ulna fracture	Surgical fixation	3
3	55	M	Trimalleolar fracture	Surgical fixation	4
4	55	M	Bimalleolar fracture	Surgical fixation	3
5	63	F	Tibial plateau, proximal fibula fracture	Knee immobilizer	8
6	21	M	C6 right pedicle fracture, L2+L3 transverse process fracture	Cervical collar	20
7	44	F	Distal fibula fracture	Controlled ankle motion boot	7
8	27	F	Distal fibula fracture	Short leg cast	8
9	21	F	Distal radius fracture, ulnar-styloid fracture	Surgical fixation	4
10	50	M	L3 superior endplate fracture	Thoracolumbosacral orthosis	14

**Table 2:** Participant oxycodone ingestion patterns from digital pills.

Participant	Pain scale		Number of doses ingested per day (pills/day)							Oxycodone dose ingested in 7 days (mg)	Continued oxycodone for pain after 7 days
	ED^a^ arrival	ED discharge	1	2	3	4	5	6	7		
1	7	7	1	2	1	1	2	4	4	75	Yes
2	9	Not recorded	2	2	3	2	2	1	0	60	Yes
3	10	Not recorded	3	7	4	0	0	0	0	70	Yes^b^
4	10	3	3	3	3	0	0	0	0	45	Yes
5	7	6	2	2	4	4	2	0	2	80	No
6	8	5	2	0	2	2	0	2	2	50	No
7	10	4	2	1	1	2	0	0	0	30	No
8	3	Not recorded	0	0	1	1	0	0	0	10	No
9	8	5	5	2	1	0	0	0	0	40	No
10	6	Not recorded	1	1	1	1	0	0	0	20	No

^a^ED: emergency department. ^b^Individual did not record continued ingestions (days 4-7) because he stopped interacting with the digital pill.

### Fidelity of the Digital Pill System

The digital pill system was able to accurately measure oxycodone ingestion events (Table 3). Participants returned the remaining digital pills at the conclusion of the study and a pill count was used to verify the fidelity of the digital pill system. A total of 96 cumulative ingestion events were recorded during the study (87.3% accuracy). A total of 14 ingestion events were not recorded by the digital pill system; all these ingestion events occurred in two participants who were unwilling to interact with the technology, stating that severe pain prevented them from using the receiver or charging the digital pill system’s batteries. Some ingestion events were initially not recorded on the cloud-based server due to lack of cellular reception at the study participant’s home. Once the receiver entered cellular service, ingestion events automatically uploaded onto the server. In these instances, the receiver was mailed to our industry partner (eTectRx) to confirm all ingestion events had been uploaded.

**Table 3:** Accuracy of the digital pill.

Participant	Ingestion events recorded	Pills taken (based on pill count)	System accuracy (%)
1	15	15	100%
2	12	12	100%
3	14	20	70%
4	9	9	100%
5	16	16	100%
6	10	18	56%
7	6	6	100%
8	2	2	100%
9	8	8	100%
10	4	4	100%
Total	96	110	87%

### Patient Response to Digital Pills

We completed open-ended interviews with all participants at the end of the study period (Textbox 1). Participants reported being able to easily operate the digital pill. Nine participants responded positively to the digital pill; one patient reported it was initially difficult for him to use the hub during the first 24 hours during episodes because of severe pain (Table 3). Attitudes toward the digital pill were positive; 80% (8/10) of participants reported that they would be willing to use the digital pill for adherence monitoring in chronic disease and would be willing to share ingestion data with physicians. Also, 90% (9/10) of participants reported they found the digital pill palatable and easy to swallow, considered the technology valuable, and had no problems operating the digital pill at home. One participant reported difficulty tolerating the size of the digital pill (a standard size 00 capsule; 8.5 mm in diameter, 23.3 mm in length; approximately the size of an 800 mg ibuprofen tablet), but reported willingness to use the digital pill.

Participants did not report any concerns regarding issues of privacy when utilizing the digital pill. Participants preferred real-time transfer of ingestion data to their physician, especially if their physician could use their ingestion data to intervene at potential times of escalating use. Interestingly, participants reported that they preferred text message-based confirmation of ingestion events and, in the context of chronic disease, would have liked mobile phone-based text messages reminding them to take medications.

**Feasibility of a digital pill.**

**Acceptance of system (9/10, 90%)**

- 90% of participants reported a positive experience integrating digital pill into medication regimen
- System became part of medication routine and participants developed different techniques for system use based on individual habits
- Digital pills were easy to swallow
- Appreciated receiving text messages after taking medication when an ingestion event was recorded
- Sample of participant response:

“I thought it was easy. It helped me, because I had a routine of just take the reader off of the charger and go get the medicine”

“I like getting the (text) message, it showed me that the device was working.”

**Willingness to use in chronic disease (8/10, 80%)**

- Participants willing to use in coordination with health care provider for observation of medication taking behavior
- Sample of participant response:

“A prompt would be nice if it was a medication I had to take daily, because people, like myself, forget to take their medication”

“Using the system actually really helped me to realize how much (oxycodone) I was taking”

2 participants reported they would not be willing to use the system in chronic disease (see barriers to use)

**Equipment failure and barriers to use (2/10, 20%)**

Problem: participant 2 lost reception due to poor cell coverage, so ingestion events were not transmitted via SMS to study server

Solution: ingestion events are stored on reader automatically for manual upload to server as backup

Problem: participant 5 reported that system was hard to remember to use when in severe pain; stopped using system on day 3

Solution: focus on patient-centered improvements for ease of use in next-generation digital pill (eg, integrating reader with receiver sticker into one easy-to-wear reader as a lanyard); use digital pill for conditions not involving severe pain

Problem: participant 5 reported improved battery life would have made system easier to use when in severe pain; participant 6 did not plug in reader when not in use causing the reader to lose power and ingestion events were not recorded on day 1 and day 2; participant 10 reported that they would not be willing to use system in chronic disease because of having to charge reader consistently

Solution: next-generation reader will have improved battery life and not need to be charged consistently when not in use

## Discussion

We demonstrated the acceptability and usability conditions of digital pills as a medication monitoring method among patients with acute painful conditions. Importantly, we successfully deployed the digital pill among ED patients; ED-based research is unique in that it oversamples poor, indigent, homeless, and immigrant populations [14]. Notably, acceptance of the digital pill allowed us to measure ingestion patterns in nontraditional research participants (ie, individuals with whom we did not have an ongoing therapeutic relationship). Therefore, digital pills may be a potential method to reinforce adherence to short regimens of high-risk medications such as opioids prescribed in the ED. Furthermore, we successfully recruited and retained participants of low socioeconomic status (including homeless individuals); we feel that our confidence in deploying digital pills in “real-world” environments is justified.

Our data show that digital pills can detect not only real-time oxycodone ingestion in real-world patients, but also the patterns by which they take these medications. Our ability to reliably measure medication ingestion events using the digital pill arises from the technology’s capacity to detect simultaneous ingestion of two separate digital pills. When two simultaneous digital pills were ingested, the digital pill recognized two distinct ingestion events 100% of the time. These pilot data demonstrate that digital pills can mirror an individual’s medication ingestion pattern in the real world and that these data can be delivered to physicians in real time.

Failure of the digital pill to record ingestion events resulted from technical difficulties of the emerging technology (eg, reader was not charged or lack of cellular signal at the reader location), not operator error. Notably, in cases when the reader could not link to a cellular network, the reader recorded ingestion events in its on-board memory, which we downloaded at participant follow-up. The 20 minutes of hands-on instruction—a time period expected to drop as technology improves—ensured fidelity to tasks needed to operate the digital pill. This short training period and 90% acceptance of the system implies that it is simple and intuitive for the user. We believe technical improvements in the reader—improved battery life, the ability to switch between wireless networks, low energy Bluetooth and cellular transmission, and improved capture of the radiofrequency signal—will only improve detection accuracy and decrease the steps required to operate the reader.

We identified conflicting responses regarding the impact of a medication monitoring system on participant behavior. For example, participants did not perceive that physician notification of real-time ingestion events altered their decisions surrounding medication use or altered the dose or frequency with which they ingested digital pills. Conversely, in the theoretical case of medication monitoring for chronic disease, participants reported that the technology would be an adherence-improving measure in itself.

Importantly, our formative interviews of study participants demonstrate their perception that the digital pill maintains patient privacy. This finding contrasts with multiple reports in the lay media hypothesizing that real-time detection of medication ingestion events could violate privacy [15,16]. Our study participants reported that our security measures—encryption of data streams, deidentification of ingestion data prior to transmission, and access of data that was limited to clinicians—were acceptable. This suggests that individuals with other stigmatized conditions that require strict medication adherence, such as substance abuse disorders or human immunodeficiency virus (HIV), may also similarly accept the data security offered through the digital pill. Continued data security and protection of patient privacy can be accomplished through excluding protected health information from ingestion data (eg, transmitting only ingestion time and pill number instead of transmitting a patient identifier) and improvements in radiofrequency encryption.

Discovering the ways in which patients take opioids in natural environments is of particular interest given the increasing rate of opioid overdose deaths. Ingestion data from our study shows that patients may require only brief periods of opioid analgesia following an acute extremity fracture. Most study participants ingested opioids for only 72 hours after their injury while self-tapering their dose. Study participants experienced greatest pain in the first 48 hours following injury. Participants successfully controlled subsequent episodes of pain with nonopioid analgesics such as acetaminophen or ibuprofen. Study participants who ingested opioids after 72 hours reported doing so for specific reasons, such as treating a second painful condition (eg, a soft tissue laceration in one case) or to maximize analgesia before bedtime. This formative data, although drawn from a small cohort of patients, may provide guidance for physicians hoping to provide effective yet safe duration of opioid therapy.

This study represents the first time digital pills have been deployed from the ED. We demonstrate that digital pills feasibly monitor medication ingestion events in the real world, require minimal training, and are acceptable by users, even those of low socioeconomic status. Our approach may be adapted to the deployment of digital pills to monitor chronic medication adherence in chronic conditions such as antiretroviral agents to prevent or treat HIV infection, or the use of anticoagulants in atrial fibrillation. Continued advances in radiofrequency signaling and energy harvesting will boost signal strength for future digital pills while continued miniaturization, improved battery life, and advanced detection techniques will allow future receivers to blend into the background of daily life, increasing acceptance and fidelity of digital pills. Automated interpretation of adherence data and integration with electronic medical records will improve data visualization by physicians and patients and may serve as a novel method to boost adherence. Digital pills may be an effective method to detect real-time medication ingestion events for many chronic, expensive, and intractable conditions.

This study had several limitations. First, our data demonstrate the feasibility of a digital pill to monitor medication ingestion, but the small sample size makes it difficult to determine whether decreased opioid usage was statistically significant. Second, we studied patients with acute extremity fracture—a painful condition for which opioids are commonly prescribed. Therefore, our data should not be generalized to all painful conditions. Future studies should focus on various painful conditions to guide the creation of a more comprehensive set of opioid-prescribing guidelines. Third, patients in this study had stringent enrollment criteria, including the lack of psychiatric illness or a history of substance abuse. Fourth, we did not follow patients after their operative repair because our main goal was to understand the feasibility and acceptability of the digital pill. Therefore, we do not know the patterns of chronic opioid use.

This pilot study demonstrates the feasibility of digital pills to detect medication ingestion events and discern patterns of opioid analgesic use in patients discharged from the ED. Patients readily accept and interact with digital pills in the real world. Our study adds to the growing data regarding natural environment interactions with digital pills. When formulated with oxycodone, digital pills can help clinicians discover natural ingestion patterns of opioids.

## Abbreviations

ED: emergency department
HIV: human immunodeficiency virus
